# Differential and Dose-Dependent Redistribution of Vitamin D Metabolites After Acute High-Intensity Exercise in Mixed Martial Arts Athletes and Untrained Men: Pilot Study

**DOI:** 10.3390/nu18071061

**Published:** 2026-03-26

**Authors:** Katarzyna Patrycja Dzik, Katarzyna Jagłowska, Miłosz Palicki, Sylwester Kujach, Piotr Sawicki, Konrad Kowalski, Jan Jacek Kaczor

**Affiliations:** 1 Department of Human and Animal Physiology, Faculty of Biology, University of Gdansk, 80-309 Gdansk, Poland; katarzyna.dzik@ug.edu.pl; 2 Division of Physiology, Gdansk University of Physical Education and Sport, 80-336 Gdansk, Poland; katarzyna.jaglowska@awf.gda.pl (K.J.); sylwester.kujach@awf.gda.pl (S.K.);; 3 Masdiag-Diagnostic Mass Spectrometry Laboratory, Stefana Żeromskiego 33, 01-882 Warsaw, Poland; konrad.kowalski@masdiag.pl

**Keywords:** vitamin D_3_, high-intensity exercise, anaerobic performance, vitamin D-binding protein, mixed martial arts

## Abstract

**Background/Objectives**: Acute high-intensity exercise may transiently alter circulating vitamin D metabolites. However, the effects of training status, supplementation dose, and vitamin D-binding protein (VDBP) on the exercise-induced redistribution and bioavailability of vitamin D remain unclear. This pilot study examined whether training status and vitamin D_3_ supplementation dose modulate vitamin D status, anaerobic performance, and acute post-exercise changes in vitamin D metabolites and VDBP. **Methods**: Thirty-four healthy young men participated, comprising 22 mixed martial arts (MMA) athletes and 12 untrained controls. MMA athletes received either 3500 or 6000 IU/day of vitamin D_3_ for four weeks, while untrained participants received 3500 IU/day or a placebo. Before and after the intervention, participants performed a supramaximal anaerobic exercise protocol (3 × 30 s Wingate tests). Blood samples were collected at rest and 30 min and 24 h post-exercise to assess vitamin D metabolites, VDBP, bioavailable and free 25(OH)D_3_, interleukin-6, and lactate. **Results**: Supplementation with 3500 IU/day significantly increased serum 25(OH)D_3_ in untrained men (*p* = 0.003) but not in MMA athletes. In contrast, 6000 IU/day increased 25(OH)D_3_ in MMA athletes to a sufficient concentration (*p* = 0.001) and improved maximal power (+7.5%), mean power (+4.9%), and total work (+5.0%). Acute exercise increased circulating vitamin D metabolites in trained athletes but reduced them in untrained men supplemented with vitamin D. **Conclusions**: The efficacy of vitamin D_3_ supplementation and the acute exercise-induced vitamin D responses appear to be training-dependent. A daily dose of 6000 IU is more effective in achieving vitamin D sufficiency and performance benefits in MMA athletes, whereas 3500 IU is sufficient for untrained men.

## 1. Introduction

Vitamin D plays a well-established role in calcium–phosphate homeostasis and skeletal health; however, its relevance in sports medicine has expanded to include skeletal muscle function, exercise adaptation, and recovery. Athletes training at high intensity, particularly at northern latitudes and during winter months, frequently present with suboptimal vitamin D status despite increased physiological demands related to repeated muscle contraction and calcium turnover. Consequently, vitamin D_3_ supplementation is commonly recommended in athletic populations; however, optimal dosing strategies and exercise-related responses remain insufficiently defined. Despite the increasing prevalence of vitamin D deficiency in athletes, research investigating the effects of vitamin D_3_ supplementation in this population remains limited. Previous studies have shown that vitamin D deficiency may increase the risk of bone injuries such as stress fractures in military and athletic populations [1,2]. Systematic reviews and meta-analyses have also demonstrated that vitamin D_3_ supplementation may improve skeletal muscle strength [3,4].

Recent evidence indicates that acute physical exercise can transiently alter the circulating concentration of vitamin D metabolites. Mosti and coworkers demonstrated that both endurance and strength exercise induce short-term changes in serum 25-hydroxyvitamin D_3_ (25(OH)D_3_) and related metabolites, suggesting that exercise itself may influence vitamin D_3_ handling rather than acting solely as a passive stressor [5]. Similarly, studies in endurance athletes have shown that prolonged and extreme exercise, such as ultramarathon running, is associated with marked post-exercise increases in circulating vitamin D metabolites, indicating the exercise-induced redistribution or mobilization of vitamin D_3_ within the body [6]. However, the magnitude and direction of these responses appear inconsistent across studies and may depend on exercise intensity, duration, baseline vitamin D status, and training level.

Importantly, most previous investigations have focused on total circulating 25(OH)D_3_, with a limited consideration of vitamin D-binding protein (VDBP) and the bioavailable and free fractions that may be more relevant to biological activity. Moreover, little is known about how training status modulates both baseline vitamin D metabolism and the acute response to high-intensity exercise, particularly in strength and combat sport athletes. Other recent research has asked not what vitamin D can do for muscle but what muscle can do for vitamin D. At somewhere between 50 and 100 days [7,8], 25(OH)D_3_ has an unusually long half-life for a steroid hormone, particularly a seco-steroid with a broken carbon ring, whereas the half-life of 1,25(OH)_2_D_3_ is more similar to other steroid hormones, at only a few days. The half-life of VDBP, which transports both the endocrine metabolite and its parent molecule, is also only a few days [9]. These characteristics of 1,25(OH)_2_D_3_, alongside its well-known dependence on UVB exposure and thus seasonal variation for vitamin D synthesis, support the existence of an extravascular storage site for vitamin D within the body [10]. Given the lipophilic nature of free vitamin D and that it is purportedly sequestered in the fat mass of obese patients, it is unlikely that the vitamin D in adipose can be mobilized and released as required into the circulation. In cell culture, radiolabeled 25(OH)D_3_ is taken up into mature myotubes but not mature adipocytes [11].

This pilot study focuses on how training status and vitamin D_3_ dose modulate the acute redistribution of vitamin D metabolites and vitamin D-binding protein following a single bout of high-intensity exercise. Therefore, this preliminary study aimed to investigate the effects of two different doses of vitamin D_3_ supplementation on vitamin D status and anaerobic performance in mixed martial arts (MMA) athletes and untrained young men. In addition, we examined the acute response of circulating vitamin D metabolites, VDBP, and vitamin D bioavailability to a single bout of high-intensity anaerobic exercise, with a particular emphasis on differences related to training status.

## 2. Materials and Methods

### 2.1. Study Design

This pilot study was conducted as a randomized, double-blind, controlled trial including two male cohorts: trained mixed martial arts (MMA) athletes and sedentary university students. A total of 34 Caucasian male participants were enrolled (22 MMA athletes and 12 students). Within the athlete group, participants were randomly assigned to receive either a high dose of vitamin D_3_ (6000 IU/day; n = 13) or a lower dose (3500 IU/day; n = 9) for four weeks. The student group was also randomly divided: 6 participants received vitamin D_3_ at 3500 IU/day, and 6 received a placebo in the form of vegetable oil.

All interventions were delivered in identical, unmarked containers to ensure blinding. Randomization and supplement allocation were performed by a researcher not involved in data collection or analysis. Randomization was conducted using a computer-generated random sequence, with allocation concealment ensured by an independent researcher. Participants and investigators remained blinded to group assignment until all analyses were completed.

Measurements were carried out at two time points: before the intervention period (Before) and immediately after the four-week intervention (After). The study protocol was approved by the Independent Bioethics Committee for Scientific Research at the Medical University of Gdańsk (approval No. NKNNB/643/2019–2020) and conducted in accordance with the Declaration of Helsinki. To ensure full methodological transparency and completeness, this study followed the guidelines outlined in the Standard Protocol Items: Recommendations for Interventional Trials (SPIRIT). This study was conducted in Gdańsk, Poland, between September and December 2022, during the late autumn and early winter months, when natural sunlight exposure was limited and endogenous vitamin D synthesis was minimal. Due to the exploratory nature of this study and the limited availability of elite MMA athletes, no a priori sample size calculation was performed. Given the small sample size, the findings should be considered exploratory and interpreted with caution. However, the group sizes were comparable to previous interventional studies conducted in trained MMA populations [12]. 

**Scheme 1:**
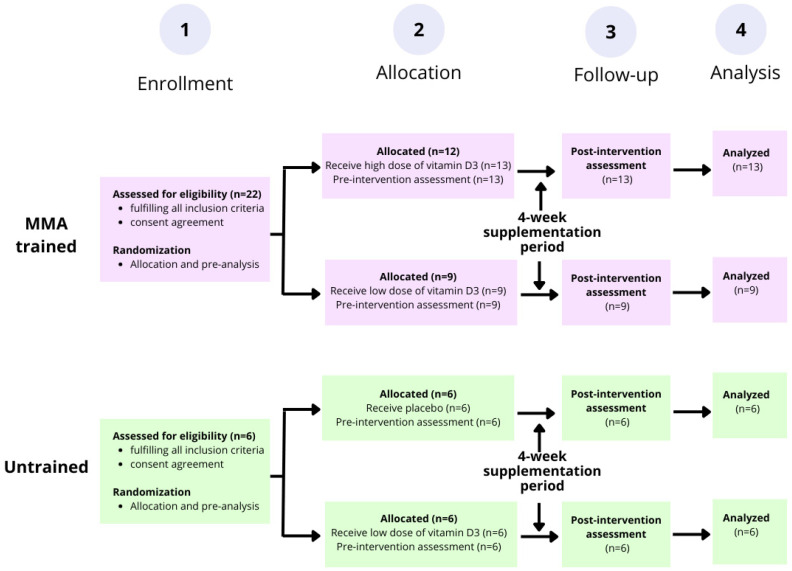
Flow diagram of participants.

This study was prospectively registered at ClinicalTrials.gov (NCT04759729) prior to participant enrollment. Although the initial registration referred specifically to MMA athletes, the protocol was subsequently extended to include a group of untrained men. Both groups were randomized and received the same supplementation protocol, and the study design and outcome measures remained consistent with the registered trial. The study design is illustrated in Scheme 1.

### 2.2. Participants

Participants were recruited from Gdańsk and the surrounding areas. Baseline characteristics are presented in Table 1. Only males aged 18 years or older were included to reduce variability related to sex and developmental stage. The athlete group consisted of individuals with several years of MMA training experience, all of whom had participated in official competitions [12]. Inclusion criteria required that athletes had no diagnosed cardiovascular diseases and no musculoskeletal injuries within the six months preceding this study. Athletes adhered to a standardized MMA training program comprising striking, grappling, strength, and endurance sessions (≥5 sessions/week, 60–90 min per session) and were instructed to maintain their habitual training and dietary practices throughout the intervention. The student group served as a non-training control cohort and did not engage in structured physical activity. This cohort was included to assess baseline vitamin D metabolism and biochemical responses in the absence of regular high-intensity training, rather than as a direct performance-matched control for elite athletes. Written informed consent was obtained from all participants after they received comprehensive information regarding the study protocol. 

### 2.3. Intervention

Vitamin D_3_ was supplemented in oil form, with a concentration of 20,000 IU per milliliter (equivalent to 0.5 mg of cholecalciferol), using Miglyol 812 as the excipient. Participants consumed the supplement once daily for four weeks at the assigned dose. Baseline assessments were conducted during the initial visit, while follow-up evaluations occurred the day after the final dose. Participants completed structured interviews before and after the intervention to document health status, medical history, medication use, dietary habits, and training routines. All participants were instructed not to introduce new supplements, alter their habitual diet, or modify physical activity patterns during this study. Training load and recovery in athletes were monitored using self-reported session duration and perceived fatigue.

### 2.4. Body Composition Analysis

Pre- and post-intervention body composition was assessed using the Tanita MC-720 segmental analyzer (multifrequency bioelectrical impedance analysis; Tanita Corporation, Tokyo, Japan). Participants stood upright during measurement, avoiding contact between the limbs and torso, while holding hand electrodes and positioning their feet on the device’s sensors. Before each measurement, individuals’ demographic data (age, sex, height) were manually input, and electrodes were cleaned with manufacturer-provided wipes to ensure accuracy. Measurements were conducted under standardized conditions (time of day and nutritional status). Outcomes included body weight, fat mass, and lean mass.

### 2.5. Blood Sampling and Biochemical Analyses

Venous blood samples were collected under standardized conditions at three time points: before exercise (baseline), 30 min post-exercise, and 24 h after the anaerobic test. All participants underwent medical screening before inclusion, and all exercise testing was supervised by qualified personnel to ensure participant safety. Blood was drawn from the arm vein into tubes containing K_2_EDTA or a coagulation activator. Following centrifugation (3000× *g*), serum and plasma were aliquoted and stored at −80 °C for further analysis.

The serum concentrations of 25(OH)D_3_, epi-25(OH)D_3_, 24,25(OH)_2_D_3_, and 25(OH)D_2_ were quantified by employing an isotope dilution technique coupled with liquid chromatography–tandem mass spectrometry (LC–MS/MS). Sample preparation and analysis were performed using the Eksigent ExionLC HPLC system equipped with a CTC PAL autosampler (CTC Analytics AG, Zwingen, Switzerland), interfaced with a QTRAP^®^ 4500 MS/MS detector (Sciex, Framingham, MA, USA). Vitamin D-binding protein (VDBP) and interleukin 6 (IL-6) concentrations were measured using a commercial ELISA kit # K2314, Immundiagnostic, Bensheim, Germany, and 950.035.192, Diaclone SAS, Besancon, France, respectively, according to the manufacturer’s instructions. Samples were processed in duplicate, and absorbance was measured at 450 nm using a microplate reader (Varioskan Flash- Spectral Scanning Multimode Microplate Reader 183, Thermo Fisher Scientific, Waltham, MA, USA). 

Subsequently, 100 µL capillary blood samples were obtained by a trained researcher during both PRE- and POST-intervention testing sessions, collected into sterile graduated microcapillary tubes. Samples were immediately transferred into microcentrifuge tubes containing 500 µL of 0.6 M perchloric acid and stored at −20 °C until subsequent analysis. Blood collection occurred before the anaerobic exercise test baseline (00) and 15, 30, and 60 min following the anaerobic exercise protocol. Lactate (LA) concentration was determined using a colorimetric kinetic assay with a Biosen C-line lactate analyzer (EKF Diagnostic, Barleben, Germany).

### 2.6. Supramaximal Interval Training

Anaerobic performance was assessed before and after the intervention in the morning following a standardized breakfast consisting of an 80 g wheat roll, 60 g strawberry jam, and one banana. Tests were conducted on a cycle ergometer (Monark 884E Sprint Bike, Monark Exercise AB, Vansbro, Sweden), with the saddle adjusted individually for each participant. The protocol began with a 5 min warm-up at 100 watts, during which two brief maximal sprints lasting 3–5 s were performed near the end. After a 3 min rest, participants completed three 30 s all-out supramaximal sprints based on the Wingate anaerobic test, with resistance set to 7.5% of body weight. Two-minute recovery intervals separated each sprint. Throughout the sprints, athletes were encouraged verbally to sustain the maximal pedaling cadence.

### 2.7. Statistical Analysis

The complete dataset from 34 participants who fully adhered to the intervention and all study protocols was analyzed. Due to the limited availability of elite MMA athletes, no a priori sample size calculation was performed, and this study was designed as exploratory. The normality of data distribution was assessed using the Shapiro–Wilk test. Descriptive statistics are presented as means with 95% confidence intervals. The baseline characteristics presented in Table 1 were analyzed using a one-way analysis of variance (ANOVA) followed by Tukey’s honestly significant difference (HSD) post hoc test when appropriate. A two-way analysis of variance (ANOVA) was used to examine the effects of group and time (as presented in the figures), followed by least significant difference (LSD) post hoc tests where appropriate. For variables assessed repeatedly across multiple time points, time was included as a within-subject factor in the ANOVA model. Baseline serum 25(OH)D concentrations were included as covariates where appropriate to account for inter-individual variability. Statistical significance was set at *p* < 0.05. All analyses were performed using Statistica software (version 13.3; StatSoft Inc., Tulsa, OK, USA).

## 3. Results

### 3.1. The Effect of 4 Weeks of Vitamin D_3_ Supplementation on the Trained and Untrained Young Men

**Figure 1:**
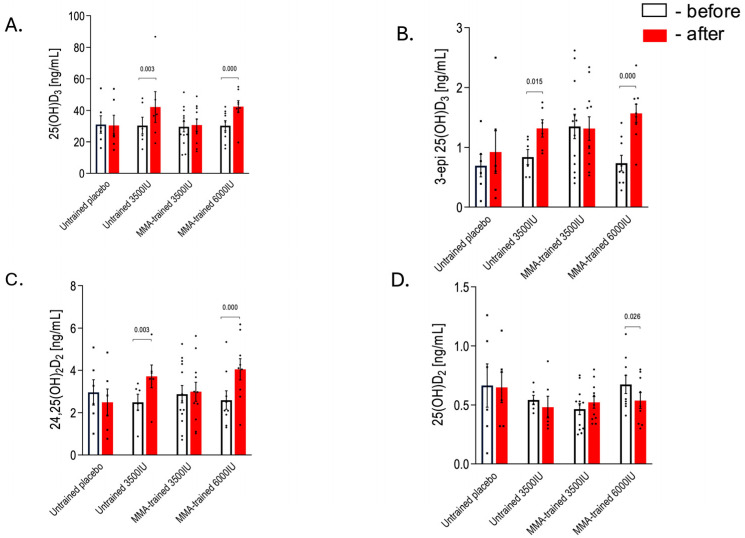
Serum vitamin D metabolites before and after supplementation. Changes in serum concentrations of 25(OH)D_3_ (**A**), 3-epi-25(OH)D_3_ (**B**), 24,25(OH)_2_D_3_ (**C**), and 25(OH)D_2_ (**D**) measured before and after supplementation in untrained-placebo, untrained–supplemented (3500 IU), MMA-trained–supplemented (3500 IU), and MMA-trained–supplemented (6000 IU) groups. Data are presented as individual values with mean ± SEM. Statistical analysis: Two-way repeated-measures ANOVA (group × time), followed by post hoc multiple-comparison testing.

Our results showed a different response to the same amount of vitamin D_3_ supplementation in MMA-trained and untrained participants. In the untrained men given 3500 IU of vitamin D_3_ for 4 weeks, the baseline 25(OH)D_3_ increased from 30.31 ± 5.34 ng mL^−1^ before supplementation to 42.11 ± 9.75 ng mL^−1^ after supplementation (*p* = 0.003), shifting the average into 25(OH)D_3_ sufficiency (>40 ng mL^−1^; Figure 1A). At the same time, supplementation with 3500 IU of vitamin D_3_ did not change serum 25(OH)D_3_ concentration in MMA athletes (29.61 ± 3.25 ng mL^−1^ before, 30.62 ± 3.88 ng mL^−1^ after). However, the baseline blood concentration in the MMA-trained athletes supplemented with 6000 IU was sufficient to shift the blood 25(OH)D_3_ concentration to a sufficient level, from 30.25 ± 3.24 ng mL^−1^ before to 42.35 ± 3.37 ng mL^−1^ after supplementation (*p* = 0.000). We did not observe a change in the baseline 25(OH)D_3_ concentration in the untrained-placebo group before and after supplementation. Due to the high demand for vitamin D [13], particularly during the winter period in Poland, and the high level of awareness among MMA athletes participating in our study, most of whom routinely take vitamin D_3_ supplements, we did not include a placebo-controlled, MMA-trained group in this study. Our goal was to observe the reaction to two different doses of vitamin D_3_ supplementation in athletes and to examine the blood concentration response of 25(OH)D_3_ to exercise. The untrained men reacted to the dose of 3500 IU; therefore, we did not see a need to supplement this group with a higher dose.

Along with the baseline change in 25(OH)D_3_, we also found a parallel, significant increase in 3-epi-25(OH)D_3_, reaching a 57% increase in the untrained-3500 IU group (*p* = 0.015) and a 113% increase in the MMA-6000 group (*p* = 0.000) and remaining unchanged in the untrained-placebo group and MMA-3500 IU (Figure 1B). Similarly, 24,25(OH)_2_D_3_ concentration also significantly rose in the same groups, reaching a 1.49-fold increase in the untrained–3500 IU group (*p* = 0.003) and 1.69-fold increase in the MMA-6000 group (*p* = 0.000) and remaining unchanged in the untrained-placebo group and MMA-3500 IU (Figure 1C). We did not observe any significant changes in 25(OH)D_2_ concentration (Figure 1D).

Collectively, these findings indicate parallel changes in 25(OH)D_3_ and its downstream metabolites, including 3-epi-25(OH)D_3_ and 24,25(OH)_2_D_3_. Only the 6000 IU regimen of vitamin D_3_ supplementation shifted trained athletes into vitamin D sufficiency (>40 ng mL^−1^) and increased metabolite concentration, supporting the need for a higher dose in high-load MMA training. The 3500 IU dose is sufficient to meet the needs of the untrained participants.

### 3.2. The Effect of 4 Weeks of Vitamin D_3_ Supplementation on Anaerobic Performance

**Figure 2:**
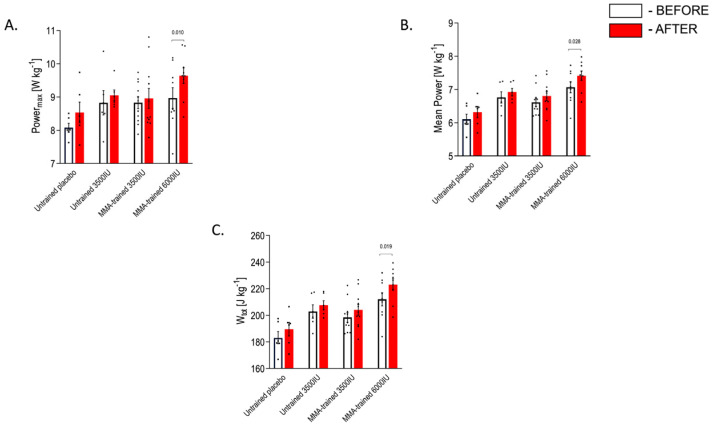
Anaerobic performance outcomes after supplementation. Peak power output (Power max, (**A**)), mean power (Mean Power, (**B**)), and total work (Wtot, (**C**)) were assessed during the Wingate anaerobic test in all study groups following the supplementation period. Data are shown as individual values with the mean ± SEM. Statistical analysis: Two-way repeated-measures ANOVA (group × time), followed by LSD post hoc multiple-comparison testing.

After the 4-week intervention, only the MMA-6000 IU group exhibited a statistically significant improvement in anaerobic performance. The maximal power (mean of three consecutive 30 s all-out trials) increased by +0.67 W kg^−1^ (+7.5%, *p* = 0.011; Figure 2A), mean power (calculated from three trials) increased by +0.35 W kg^−1^ (+4.9%, *p* = 0.028; Figure 2B), and mean total work (three consecutive 30 s all-out trials) increased by +10.4 J kg^−1^ (+5.0%, *p* = 0.019; Figure 2C). Taken together, the consistent improvements in maximal power, mean power, and total work provide converging evidence that the observed increase in serum 25(OH)D_3_ concentration (+12 ng mL^−1^, reaching a sufficient level) is associated with enhanced anaerobic capacity in trained athletes.

The 3500 IU-dose MMA cohort exhibited uniform but non-significant improvements: mean total work, +2.8% (n.s.); mean power, +2.9% (n.s.); and peak power, +1.4% (*p* = 0.46). Among untrained participants in both the 3500 IU and placebo groups, small increases were observed across all three parameters; however, none reached statistical significance. In summary, supplementation with 6000 IU vitamin D_3_ in MMA-trained young men was the only condition that elicited a statistically significant improvement in peak anaerobic performance parameters.

Data from each of the three bouts of supramaximal sprints performed during the WAnT are provided in the Supplementary File, with Table S1 presenting the mean power, Table S2 total work, Table S3 maximal power, and Table S4 time to maximal power.

### 3.3. Acute 25(OH)D_3_ Response to High-Intensity Anaerobic Exercise

**Figure 3:**
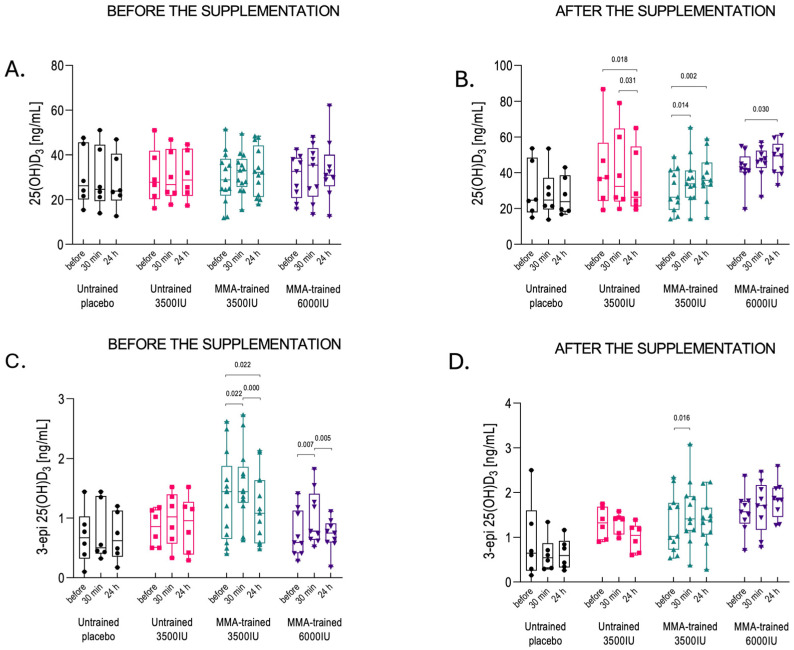
Acute exercise-induced changes in 25(OH)D_3_ and 3-epi-25(OH)D_3_. Serum concentrations of 25(OH)D_3_ before (**A**) and after supplementation (**B**) and 3-epi-25(OH)D_3_ before (**C**) and after supplementation (**D**), measured at baseline and 30 min and 24 h after exercise in all study groups. Data are presented as box-and-whisker plots showing median and interquartile range (box: 25th–75th percentile), with whiskers indicating minimum and maximum values. Individual data points are displayed for each participant. Statistical analysis: Two-way repeated-measures ANOVA (group × time), followed by LSD post hoc multiple-comparison testing.

Before supplementation (Figure 3A), only the MMA cohorts exhibited a slight rise in serum 25(OH)D_3_ following exercise. The MMA-3500 IU group showed an increase of 25(OH)D_3_ by +2.32 ng mL^−1^ at 30 min post-exercise (+8%, n.s.), which remained elevated by 2.84 ng mL^−1^ up to 24 h after exercise (+10%, n.s.) compared with baseline. The MMA-6000 group displayed a smaller, non-significant increase of 25(OH)D_3_ +1.9 ng mL^−1^ at 30 min (+6.4%), which continued to rise over the next 24 h, reaching +3.06 ng mL^−1^ (+10%, n.s.) above baseline. In contrast, untrained groups demonstrated a decreasing trend in 25(OH)D_3_ concentration 30 min post-exercise, but this difference was not significant.

After four weeks of vitamin D_3_ supplementation (Figure 3B), this pattern became more pronounced. The MMA-3500 group exhibited a +5.46 ± 0.9 ng mL^−1^ increase at 30 min (+17.8%, *p* = 0.014) relative to baseline, with an even greater elevation after 24 h: +6.96 ng mL^−1^ (+22.7%, *p* = 0.002). The MMA-6000 group maintained a moderate increase of +3.72 ng mL^−1^ (+5.2%, n.s.) at 30 min post-exercise, which rose to +5.28 ng mL^−1^ (+12%, *p* = 0.030) at 24 h compared with baseline.

In contrast, the untrained-3500 IU group experienced a marked decline at 24 h after exercise, with a decrease of −7.07 ng mL^−1^ (−16.8%, *p* = 0.018) from baseline, accentuating the downward trend observed before supplementation. This effect was observed only in the untrained group supplemented with 3500 IU of vitamin D_3_. In the placebo–untrained group, we did not observe any alteration. 

These findings indicate that vitamin D_3_ supplementation modulates the acute post-exercise 25(OH)D_3_ response in a dose- and training status-dependent manner, with pronounced increases observed only in MMA-trained athletes following supplementation and a notable post-exercise decline in untrained individuals receiving 3500 IU. The mechanism underlying this decline remains unclear and warrants further investigation.

### 3.4. Acute 3-epi-25(OH)D_3_ Response to High-Intensity Anaerobic Exercise

Before supplementation (Figure 3C), the immediate response of 3-epi-25(OH)D_3_ to acute exercise in MMA-trained young men was similar to the 25(OH)D_3_ fluctuations, with an increase observed 30 min post-exercise. However, while 25(OH)D_3_ concentration remained stable after 24 h, 3-epi-25(OH)D_3_ showed a significant decline. In the MMA-trained 3500 IU group, we observed a significant increase of 3-epi-25(OH)D_3_ by 0.20 ng mL^−1^ (+14.66%, *p* = 0.022) compared with the baseline measurement. After 24 h, this value dropped significantly by 0.20 ng mL^−1^ (−14.6%, *p* = 0.022) compared with the baseline and by 0.40 ng mL^−1^ (−25.5%, *p* = 0.000) compared with the 30 min post-exercise concentration. In the MMA-trained–6000 IU group, 3-epi-25(OH)D_3_ increased by 0.28 ng mL^−1^ (+38.07%, *p* = 0.007) compared with baseline, followed by a decline of 0.30 ng mL^−1^ in 3-epi-25(OH)D_3_ after 24 h in comparison to the 30 min post-exercise level (−41%, *p* = 0.006). Untrained groups showed no significant fluctuations in 3-epi-25(OH)D_3_.

These results demonstrate that, although the immediate post-exercise response in MMA-trained athletes paralleled the fluctuations observed for 25(OH)D_3_, the 3-epi-25(OH)D_3_ metabolite exhibited a distinct pattern, with significant short-term elevations followed by marked declines within 24 h—a trend absent in untrained individuals.

After four weeks of vitamin D_3_ supplementation (Figure 3D), the response pattern changed. The MMA-3500 group still exhibited a significant acute rise in 3-epi-25(OH)D_3_ by +0.23 ng mL^−1^ (+18% at 30 min; *p* = 0.016), but the concentration of 3-epi-25(OH)D_3_ measured 24 h post-exercise returned to baseline concentration (n.s.). At the same time, the MMA-6000 IU group showed no significant increases in 3-epi-25(OH)D_3_ concentration at either time point. In the untrained-3500 IU group, we observed a decreasing trend at 24 h post-exercise: −25.62% compared with the resting state and −23.58% compared with the 30 min post-exercise level. The placebo–untrained group showed no significant changes.

These findings suggest that vitamin D_3_ supplementation modifies the acute 3-epi-25(OH)D_3_ response in a manner dependent on both training status and dose, with notable post-exercise fluctuations persisting only in MMA-trained individuals and absent in untrained participants. Additionally, Supplementary Figure S1 presents the acute changes in 24,25(OH)_2_D_3_.

### 3.5. Acute VDBP and Vitamin D_3_ Bioavailability Response to High-Intensity Anaerobic Exercise

**Figure 4:**
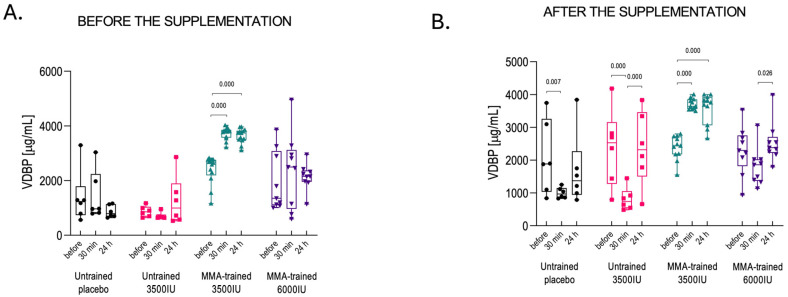
Vitamin D-binding protein (VDBP) response to exercise. Serum vitamin D-binding protein (VDBP) concentrations were measured before (**A**) and after supplementation (**B**) at baseline and 30 min and 24 h post-exercise in all experimental groups. Data are presented as box-and-whisker plots showing the median and interquartile range (box: 25th–75th percentile), with whiskers indicating the minimum and maximum values. Individual data points are displayed for each participant. Statistical analysis: Two-way repeated-measures ANOVA (group × time), followed by post hoc multiple-comparison testing.

Regarding MMA-trained groups, we observed an increase in the VDBP level but only in the MMA-3500 IU group. This change was significant at both 30 min (+55%, *p* = 0.000) and 24 h (+51%, *p* = 0.000) after exercise compared with the baseline level. At the same time, in the untrained groups, the VDBP level showed only slight fluctuations (Figure 4A). 

After supplementation, an increase in VDBP was registered in the MMA-3500 IU group (+56%, *p* = 0.000) 30 min after the WAnT as compared to the baseline level (Figure 4B). Importantly, this increase was maintained at a similar level 24 h after exercise (+51%, *p* = 0.000). At the time, the observed fluctuations in the MMA-6000 IU group did not match those in the MMA-3500 IU group. A total of 30 min after exercise, the VDBP level initially dropped by 492 µg mL^−1^, and then it significantly rose by 738 µg mL^−1^ (+41%, *p* = 0.026) as compared to the 30 min post-exercise level. Surprisingly, we observed a significant decline in the VDBP level in both the untrained groups. In the untrained-placebo group, the VDBP level dropped by 878 µg mL^−1^ (−47%, *p* = 0.007), while in the untrained-3500 IU group, it dropped by 1567 µg mL^−1^ (−66%, *p* = 0.000) as compared to the baseline level. These changes may partially result from exercise-induced plasma volume shifts; however, consistent group-specific patterns suggest a regulated response rather than just a hemodilution effect.

Vitamin D in the circulation is predominantly bound to specific plasma proteins. Approximately 85–90% of both 25(OH)D_3_ and 1,25(OH)_2_D_3_ are bound to VDBP, while about 10–15% of 25(OH)D_3_ is bound to albumin. Only a very small fraction, around 1%, remains unbound (free) [14]. Because albumin has a much lower affinity for vitamin D metabolites compared with VDBP, the fraction loosely bound to albumin, together with the free fraction, is collectively defined as bioavailable vitamin D [15]. In other words, free 25(OH)D_3_ refers specifically to the unbound molecules circulating, whereas bioavailable 25(OH)D_3_ encompasses both this free fraction and the albumin-bound fraction, which can readily dissociate and enter target tissues.

**Figure 5:**
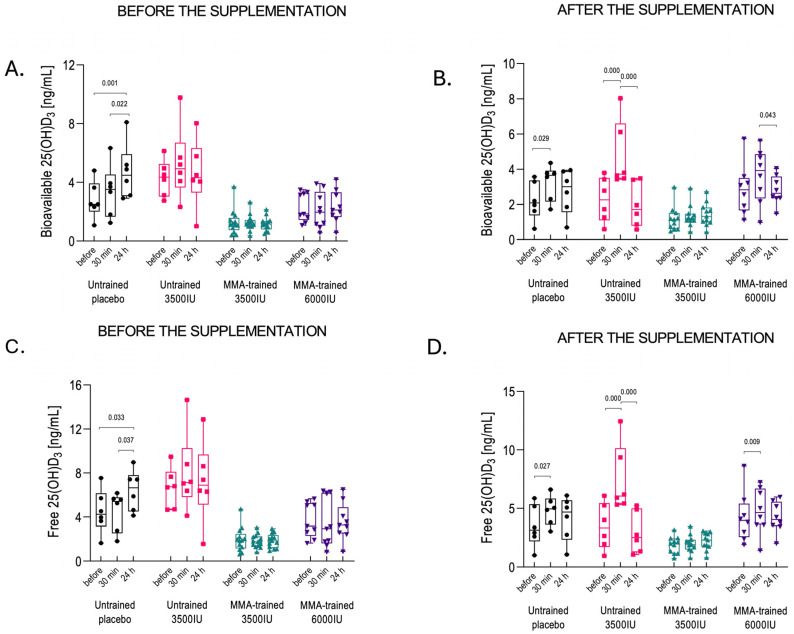
Bioavailable and free 25(OH) D_3_ before and after supplementation. Bioavailable 25(OH)D_3_ before (**A**) and after supplementation (**B**) and free 25(OH)D_3_ before (**C**) and after supplementation (**D**), measured at baseline and 30 min and 24 h post-exercise in all groups. Data are presented as box-and-whisker plots showing the median and interquartile range (box: 25th–75th percentile), with whiskers indicating the minimum and maximum values. Individual data points are displayed for each participant. Statistical analysis: Two-way repeated-measures ANOVA (group × time), followed by post hoc multiple-comparison testing.

We observed different trends in the fluctuation in the bioavailable and free 25(OH)D_3_ acute response to exercise between untrained and MMA-trained men, as well as before and after supplementation (Figure 5). While in both untrained groups before supplementation, bioavailable and free 25(OH)D_3_ increased, at the same time in the MMA-trained men, it decreased. In the untrained-placebo group, the bioavailable 25(OH)D_3_ increased by 0.59 ng mL^−1^ (+21% to the baseline) 30 min after exercise and by an additional 1.32 ng mL^−1^ 24 h after exercise (+39% compared to 30 min post-exercise, *p* = 0.022) (Figure 5A). After supplementation in the untrained-placebo group 30 min post-exercise, bioavailable 25(OH)D_3_ increased by 1.03 ng mL^−1^ (+47%, *p* = 0.029) as compared to the baseline concentration (Figure 5B). At the same time, in the untrained-3500 IU group, the increase was even more prominent, and the 30 min post-exercise concentration of bioavailable 25(OH)D_3_ was 2.1-fold higher than the baseline (*p* = 0.000), if fully normalized 24 h after exercise. In the MMA-3500 IU group, we did not observe any difference in the bioavailability of 25(OH)D_3_. In the MMA-6000 IU group, we found a substantial increase of 0.78 ng mL^−1^ 30 min after exercise (+27%, *p* = 0.043) compared to baseline.

Similar trends were observed in the free 25(OH)D_3_ concentration, before and after supplementation. Before supplementation in the untrained-placebo group, 24 h after exercise, the free 25(OH)D_3_ level rose by 1.89 pmol mL^−1^ (+42%, *p* = 0.033) as compared with the baseline level (Figure 5C). After supplementation, in the same group, a peak was observed sooner; 30 min after exercise, the free 25(OH)D_3_ level rose by 1.33 pmol mL^−1^ (+38%, *p* = 0.027; Figure 5D). In the untrained group supplemented with 3500 IU of vitamin D_3_, the free 25(OH)D_3_ level rose by 3.49 pmol mL^−1^ (+114%, *p* = 0.000) compared to the baseline. Similarly to the observation in bioavailable 25(OH)D_3_, among the MMA-trained group, only in the athletes supplemented with 6000 IU did the free 25(OH)D_3_ rise by 1.38 pmol mL^−1^ (+38%, *p* = 0.009).

### 3.6. IL-6 Response to High-Intensity Anaerobic Exercise

**Figure 6:**
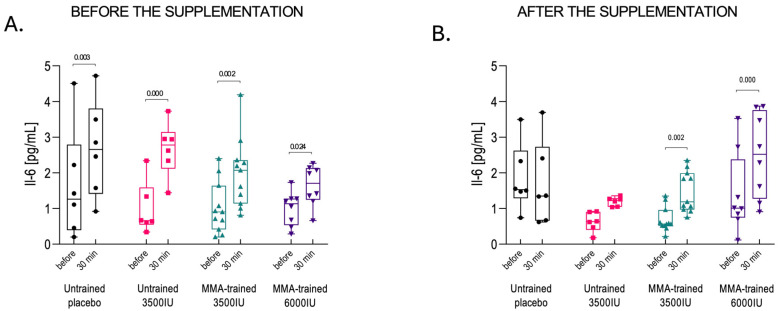
Interleukin-6 (IL-6) response to exercise. Serum IL-6 concentrations measured before (**A**) and after supplementation (**B**) at baseline and 30 min post-exercise in all study groups. Data are presented as box-and-whisker plots showing the median and interquartile range (box: 25th–75th percentile), with whiskers indicating the minimum and maximum values. Individual data points are displayed for each participant. *p*-values were obtained using a two-way repeated-measures ANOVA (group × time), followed by LSD post hoc multiple-comparison testing.

IL-6 followed the classic exercise-elicited “spike-and-return” pattern (Figure 6). Before supplementation (Figure 6A), all groups presented a significant increase in IL-6. After supplementation, the same trend was observed in all groups, except in the untrained-placebo group, where IL-6 rose significantly before supplementation (+1.02 pg mL^−1^; +61.8%, *p* = 0.003), but no significant post-exercise change was observed after the intervention (Figure 6B).

Before supplementation, in the MMA-3500 IU group, IL-6 increased by +0.97 pg mL^−1^ (+95.3%, *p* = 0.002), while in the MMA-6000 IU group, the increase reached +0.93 pg mL^−1^ (+93.0%, *p* = 0.024). After four weeks of vitamin D_3_ supplementation, the acute IL-6 response persisted. In the MMA-3500 IU group (+0.78 pg mL^−1^; +113.9%, *p* < 0.001), compared to MMA-trained athletes receiving 6000 IU, the post-exercise level of IL-6 was increased to +1.08 pg mL^−1^ (+77%, *p* = 0.000).

### 3.7. LA Changes Correlate with Vitamin D_3_ Changes

**Figure 7:**
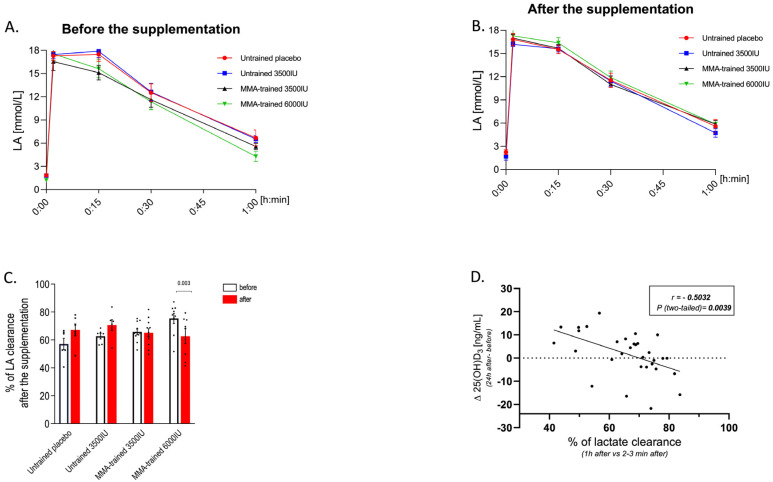
Blood lactate kinetics and association with vitamin D response. Blood lactate concentration over 60 min of recovery before (**A**) and after supplementation (**B**) in all study groups. The percentage of lactate clearance calculated as the relative decrease from peak lactate concentration (2–3 min post-exercise) to 60 min post-exercise before and after the supplementation (**C**). The association between the percentage of lactate clearance and change in serum 25(OH)D_3_ concentration (24 h post-exercise minus baseline) is shown in panel (**D**). *p*-values were obtained using a repeated-measures ANOVA (group × time), followed by an LSD post hoc test. Data in panels A and B are presented as the mean ± SEM. Data in panel C are presented as individual values with the mean ± SEM. Data for panel D are presented as Pearson correlation coefficients (r).

Before supplementation (Figure 7A), blood lactate (LA) concentration increased rapidly following the WAnT in all groups, reaching peak values 2–3 min post-exercise, followed by a progressive decline at 15, 30, and 60 min. After four weeks of vitamin D_3_ supplementation (Figure 7B), the overall temporal pattern of LA accumulation and clearance changed. In the untrained participants, LA clearance kinetics showed no consistent improvement after supplementation. Similarly, no change in the percentage of LA clearance was observed in the MMA-3500 IU group (Figure 7C). In contrast, the MMA-6000 IU group exhibited a significantly lower percentage of LA clearance (*p* = 0.003).

### 3.8. Correlation Between LA Clearance and Changes in 25(OH)D_3_

After four weeks of vitamin D_3_ supplementation, a significant relationship was observed between LA clearance and the acute exercise-induced change in serum 25(OH)D_3_ (Figure 7D). The percentage decrease in blood LA concentration from peak values (2–3 min post-exercise) to 60 min of recovery was negatively correlated with the post-exercise increase in 25(OH)D_3_, indicating that individuals exhibiting a greater rise in circulating 25(OH)D_3_ demonstrated a less pronounced LA decline during recovery (r = −0.5032; *p* = 0.038). Before supplementation, no significant association was detected between LA clearance parameters and exercise-induced changes in 25(OH)D_3_ (n.s.)

## 4. Discussion

Here, we demonstrate that the same dose of vitamin D_3_ given to either trained athletes or untrained participants for 28 consecutive days drives different responses. While in untrained men, this dose was sufficient to shift vitamin D status into the sufficiency range, in MMA-trained athletes under winter conditions, it appeared insufficient. However, supplementation with 6000 IU/day effectively increased serum 25(OH)D_3_ concentration into the sufficiency range in MMA-trained athletes without any observed adverse effects. This finding is consistent with previous studies suggesting that athletes may require higher vitamin D_3_ supplementation in certain conditions [16]. Supplementation of between 800 IU and 1000–2000 IU/day is recommended to maintain status for the general population. To date, no athlete-specific recommendations have been established for vitamin D_3_ intake or supplementation.

The present study confirms that seasonal factors, such as winter, and geographic latitude (52° N) are associated with the necessity for vitamin D_3_ supplementation in northern European countries. A total of 50% of the athletes participating in this study were vitamin D-insufficient (serum 25(OH)D_3_ below 30 ng). This reflects the higher prevalence of vitamin D inadequacy in athletes found by Farrokhyar and coworkers [17] in a meta-analysis of 2313 international participants. The authors calculated that athletes who lived above 40° N had a 1.85 times higher risk ratio (95% CI, 1.25–2.53) for vitamin D inadequacy than athletes living further south. Supplementation should be considered for athletes living at UVB-deficient latitudes, especially in the winter months.

In our previously published study, the vitamin D_3_ supplementation of MMA athletes at a dose of 3500 IU/day for 4 weeks was not sufficient to increase the serum 25(OH)D_3_ concentration above 30 ng mL^−1^ [12]. A study conducted by Owens et al. [18] on forty-six male elite professional athletes showed that a dose of 35,000 or 70,000 IU per week (which are respectively 5000 and 10,000 IU per day) led to an increase in serum 25(OH)D_3_ above 160 nmol L^−1^ (64 ng mL^−1^). Importantly, the authors showed that a dose of 70,000 IU per week led to an increase in the vitamin D metabolite 24,25(OH)_2_D_3_, which was previously shown to limit the transcriptional activity of active vitamin D 1,25(OH)_2_D_3_. In our data, we also observed an increase in 24,25(OH)_2_D_3_ in the MMA-6000 IU and untrained-3500 IU groups. The conversion of 25(OH)D_3_ to 24,25(OH)_2_D_3_ may indicate the activation of vitamin D catabolic pathways, primarily mediated by CYP24A1, which is known to be upregulated when circulating vitamin D concentration increases. An elevation in 24,25(OH)_2_D_3_ may therefore indicate the enhanced turnover of vitamin D metabolites in response to supplementation. Alternatively, increased 24,25(OH)_2_D_3_ concentrations may reflect the greater overall activity of the vitamin D endocrine system, as downstream metabolism is closely linked to the 1,25(OH)_2_D_3_-dependent regulation of *CYP24A1* expression. Although the physiological role of 24,25(OH)_2_D_3_ is not yet fully understood, its accumulation is commonly interpreted as a marker of increased vitamin D metabolic flux. Nonetheless, the most interesting result is the lack of serum increase in both 25(OH)D_3_ and 24,25(OH)_2_D_3_ in MMA-3500 IU, which suggests that in MMA-trained athletes, a daily dose of 3500 IU may primarily support the ongoing metabolic conversion or tissue distribution of vitamin D metabolites, rather than increasing circulating 25(OH)D_3_ concentrations.

The effect of vitamin D_3_ supplementation on strength and power appears to be inconsistent and is heavily influenced by the type of training that is undertaken in each study. The inconsistency in the effect of vitamin D on anaerobic strength/power and sprint speed among the included studies is likely owed to the variability in training modality and sport-specific metabolic demands. A previous systematic review and meta-analysis of 310 young and active participants concluded that vitamin D supplementation was associated with increased upper (*p* = 0.005) and lower (*p* = 0.04) body strength [3].

Our study shows that only the higher dose of vitamin D_3_ (6000 IU/day) led to significantly increased mean power, total work, and maximal power during 3xWAnT in MMA athletes. Although the current study was not specifically designed to assess dose–response relationships, the observed differences between the 3500 IU and 6000 IU groups suggest a potential dose-dependent effect of vitamin D_3_ supplementation. Interestingly, although the untrained males also exhibited an increase in serum 25(OH)D_3_ concentration, this did not lead to better outcomes of 3xWAnT. This suggests that vitamin D-related adaptations may interact with training-induced muscular adaptations, rather than exerting uniform effects across different training statuses.

The current study underscores the importance of assessing training status in vitamin D research. Here, we observed a tremendous difference between trained and untrained men in serum vitamin D metabolite responses upon a single bout of high-intensity exercise (3xWAnT). In MMA athletes, serum 25(OH)D_3_ and 3-epi-25(OH)D_3_ concentrations increased significantly at both 30 min and 24 h post-exercise. It should be noted that high-intensity exercise may cause acute reductions in plasma volume, leading to hemoconcentration and, consequently, changes in circulating biomarker levels. Therefore, the observed post-exercise increases in 25(OH)D_3_ and related parameters may, at least in part, reflect changes in plasma volume rather than true metabolic alterations. However, the pattern and magnitude of changes suggest that physiological mechanisms, such as the exercise-induced redistribution or mobilization of vitamin D metabolites, cannot be excluded. Importantly, this response was observed regardless of supplementation and vitamin D_3_ dose. However, the deficient athletes (MMA-3500 IU) had the highest increase in circulating 25(OH)D_3_. In contrast, in untrained participants, we detected a decrease in both 25(OH)D_3_ and 3-epi-25(OH)D_3_ but only after 4 weeks of vitamin D_3_ supplementation at a dose of 3500 IU/day.

Vitamin D C-3 epimerization has been shown to occur in multiple tissues, including the liver and kidneys, and may be transiently enhanced during acute exercise as a consequence of increased metabolic turnover and enzymatic activity within classical vitamin D metabolic pathways [19]. The exercise-induced changes in circulating 3-epi-25(OH)D_3_ observed in MMA-trained athletes indicate that this metabolite responds dynamically to acute metabolic stress, showing a transient post-exercise elevation followed by rapid normalization within 24 h. Previous studies have demonstrated that 3-epi-25(OH)D_3_ closely follows alterations in 25(OH)D_3_ availability and reflects changes in vitamin D metabolic handling rather than classical vitamin D receptor-mediated activity [20]. In this context, the presence of pronounced post-exercise fluctuations exclusively in trained athletes, and their attenuation after supplementation suggests that 3-epi-25(OH)D_3_ may represent a marker of increased short-term vitamin D metabolic turnover associated with elevated metabolic demand, rather than a direct indicator of vitamin D biological action or oxidative stress.

These preliminary results suggest that the high-intensity bout of exercise may represent a strong stimulus for the uptake of 25(OH)D_3_ by other tissues. Since in highly trained athletes, we found the opposite trafficking pattern of 25(OH)D_3_, we suppose that in untrained men, 25(OH)D_3_ is taken up by the tissues activated during exercise, such as skeletal muscles or adipose tissue. These findings are consistent with the hypothesis that skeletal muscle may contribute to vitamin D handling in trained individuals. Repeated high-intensity training may promote tissue-level adaptations that influence the redistribution of circulating 25(OH)D_3_ in response to acute exercise. To our knowledge, this is the first study to demonstrate that circulating vitamin D metabolites respond differently to a single bout of exercise depending on training status.

A study conducted on rats showed the increased expression of vitamin D receptor (VDR) and 1-alpha hydroxylase in skeletal muscle after a single bout of resistance exercise, an effect not observed after endurance exercise [21], indicating that exercise type and intensity are critical determinants of vitamin D metabolism in skeletal muscle. In our previous study on prepubertal and pubertal trained boys, we evaluated the effect of the acute WAnT and maximal oxygen uptake (VO_2max_) tests, representing high-intensity interval and endurance exercise, respectively. The results demonstrated that not only the type of exercise affects 25(OH)D_3_ serum alteration but also puberty status. Namely, postpubertal boys (evaluated against the appropriate weight and height percentile grids developed by the Mother and Child Institute, Warsaw, Poland) showed the most pronounced rise in serum 25(OH)D_3_ concentration after the WAnT. Importantly, in the group of postpubertal boys, the free fatty mass (FFM) was significantly higher than that in the prepubertal group of boys, suggesting that muscle mass plays a substantial role in serum 25(OH)D_3_ fluctuations. Moreover, the delta change in 25(OH)D_3_ before and 15 min after exercise correlated with FFM in all boys [22].

Similarly, in the current study, we observed pronounced changes in circulating VDBP, which may substantially contribute to fluctuations in vitamin D metabolites. Although 25(OH)D_3_ can pass into and out of muscle cells unaided, VDBP has been shown to cross the muscle cell membrane in the presence of megalin, a member of the low-density lipoprotein receptor family [23]. VDBP was shown to bind to intracellular skeletal muscle actin, as previously reported in hepatocytes [24]. Thus, vitamin D can be stored in muscle as actin-bound VDBP, which may be proteolyzed to free 25(OH)D_3_, back into the circulation [25]. Importantly, exercise-induced alterations in VDBP concentration may directly influence the proportion of free and bioavailable vitamin D, suggesting that changes in total circulating 25(OH)D_3_ do not necessarily reflect the biologically active fraction, particularly in trained individuals exposed to repeated high-intensity exercise.

In the present study, the exercise-induced alterations in vitamin D metabolites occurred in parallel with marked metabolic and endocrine responses to high-intensity exercise. Notably, we detected a negative association between circulating vitamin D metabolites and markers of metabolic stress, including LA accumulation and IL-6 response. Given that both IL-6 and LA are key exerkines released during intense anaerobic exercise and act as systemic signals regulating hepatic metabolism and substrate redistribution, their elevation reflects a pronounced metabolic response to exercise. The observed inverse relationship, therefore, suggests that increased exerkine signaling may be accompanied by a transient shift in vitamin D metabolite handling rather than indicating a deleterious effect; instead, this pattern likely reflects an integrated adaptive response to acute metabolic demand, whereby vitamin D metabolites are dynamically redistributed or metabolized in concert with exercise-induced exerkine signaling.

## 5. Conclusions

In conclusion, the vitamin D flow is dependent on training status, and athletes have different needs for vitamin D_3_ supplementation doses. It is interesting that in untrained young men supplemented with 3500 IU daily, we observed an increase in serum 25(OH)D_3_ concentration, whereas the same dose given to trained men showed no difference. Furthermore, in untrained men, high-intensity training caused a decrease in 25(OH)D_3_ within 24 h after exercise, whereas trained athletes did not experience this decline and instead showed an increase in 25(OH)D_3_ concentration. This suggests that MMA-trained athletes may have already undergone training adaptation, which leads to the accumulation of vitamin D. As previous studies show, it may be accumulated in both adipose and muscle tissues. With the 3500 IU dose, it is possible that at this dosage, vitamin D_3_ is preferentially metabolized or redistributed to peripheral tissues, limiting detectable increases in circulating 25(OH)D_3_, whereas the 6000 IU dose is sufficient for the maintenance of temporary needs for daily training, which often includes high-intensity workouts for highly trained athletes. It is also accumulated in sufficient amounts in this case; therefore, we observed an increase in serum 25(OH)D_3_ concentration.

At the same time, we need to address the lower percentage of fat in body composition. The MMA-trained athletes have significantly lower fat mass and higher FFM compared with untrained men, indicating that accumulation and release may be primarily dependent on muscle mass. Taken together, these findings suggest that vitamin D trafficking and utilization are strongly dependent on training status and body composition. The higher FFM and lower fat mass observed in MMA-trained athletes support the concept that skeletal muscle may represent a primary site of vitamin D storage and release during repeated high-intensity training. In contrast, in untrained individuals, the acute post-exercise decrease in circulating 25(OH)D_3_ and 3-epi-25(OH)D_3_ may reflect a transient redistribution of vitamin D metabolites toward metabolically active tissues during exercise-induced stress. Importantly, vitamin D may play a role in exercise adaptation that is likely mediated through the modulation of metabolic and inflammatory pathways, including the regulation of enzymes involved in redox homeostasis. Therefore, the divergent post-exercise responses observed between trained and untrained participants likely reflect differences in the metabolic adaptation and efficiency of vitamin D handling.

*Limitations of this Study*

A limitation of the present study is that exercise-induced changes in 25(OH)D_3_ were not adjusted for changes in plasma volume, which may have influenced post-exercise concentrations. Previous research has shown that acute exercise may alter plasma volume and potentially influence the apparent concentrations of circulating biomarkers, including vitamin D metabolites [5]. Therefore, the observed post-exercise fluctuations in serum 25(OH)D_3_ should be interpreted as apparent concentration changes rather than direct evidence of altered vitamin D production or release. Due to the difficulty in recruiting elite MMA athletes, the present study should be considered exploratory in nature. Therefore, the findings should be interpreted with caution, particularly given the relatively small sample size. Moreover, another limitation of this study is the absence of a placebo group among trained athletes, due to ethical concerns regarding winter vitamin D_3_ supplementation. Despite these limitations, this study provides clinically relevant data from a well-defined athletic population, addressing both supplementation- and exercise-related aspects of vitamin D metabolism under real-world training conditions.

## Figures and Tables

**Table 1:** Participant characteristics.

Participant Information	Untrained-Placebo	Untrained-3500 IU	MMA-Trained-3500 IU	MMA-Trained-6000 IU	*p*-Value (Global)	Post Hoc *p*-Value
Height (cm)	178.2 ± 4.3	176.7 ± 6.2	179.3 ± 7.7	179.1 ± 7.6	0.885	-
Weight (kg)	81.5 ± 6.9	75.6 ± 9.9	80.2 ± 9.8	79.4 ± 11.9	0.753	-
FFM (kg)	67.1 ± 3.0 * ^c,d^	65.0 ± 7.5	71.9 ± 8.2 * ^a^	71.1 ± 8.4 * ^a^	0.004 *	^a^ vs. ^c^ = 0.006 ^a^ vs. ^d^ = 0.008
FFM (%)	82.7 ± 6.0	86.1 ± 2.6	89.8 ± 2.9	89.9 ± 3.7	0.245	-
FM (kg)	14.4 ± 5.8 * ^c,d^	10.6 ± 3.1	8.3 ± 2.78 * ^a^	8.4 ± 4.3 * ^a^	0.004 *	^a^ vs. ^c^ = 0.007 ^a^ vs. ^d^ = 0.008
FM (%)	17.3 ± 6.0 * ^c,d^	13.9 ± 2.7	10.3 ± 3.1 * ^a^	10.1 ± 3.7 * ^a^	0.025 *	^a^ vs. ^c^ = 0.026 ^a^ vs. ^d^ = 0.036

Values are presented as the mean ± SD. The global *p*-value refers to the overall comparison among all four groups and was determined using a one-way ANOVA. Tukey’s HSD post hoc test was applied. Values marked with asterisks (*) indicate statistically significant differences (*p* < 0.05). Superscript letters indicate significant pairwise differences between groups: ^a^—untrained-placebo; ^c^—MMA-trained-3500 IU; ^d^—MMA-trained-6000 IU. FFM, fat-free mass; FM, fat mass.

## Data Availability

The data presented in this study are available on request from the corresponding author, as they are not publicly available due to privacy or ethical restrictions.

## Supplementary Materials

The following supporting information can be downloaded at https://www.mdpi.com/article/10.3390/nu18071061/s1, Figure S1: Acute exercise-induced changes in 24,25(OH)2D3; Table S1: Mean power [W·kg^−1^] obtained during Supramaximal Sprints; Table S2: Total work [J·kg^−1^] obtained during Supramaximal Sprints; Table S3: Maximal power [W·kg^−1^] obtained during Supramaximal Sprints; Table S4: Time to maximal power [s] obtained during Supramaximal Sprints.

## Abbreviations

The following abbreviations are used in this manuscript:

1,25(OH)_2_D_3_	1,25-dihydroxyvitamin D_3_
24,25(OH)_2_D_3_	24,25-dihydroxyvitamin D_3_
25(OH)D_3_	25-hydroxyvitamin D_3_ (25-hydroxycholecalciferol)
3-epi-25(OH)D_3_	3-epi-25-hydroxyvitamin D_3_ (3-epimer of 25-hydroxyvitamin D_3_)
CYP24A1	Cytochrome P450 family 24 subfamily A member 1(vitamin D 24-hydroxylase)
FFM	Fat-free mass
IL-6	Interleukin-6
IU	International units
LA	Lactate
LSD	Least significant difference
MMA	Mixed martial arts
MP	Mean power
Pmax	Maximal power
VDBP	Vitamin D-binding protein
VDR	Vitamin D receptor
VO_2max_	Maximal oxygen uptake
WAnT	Wingate anaerobic test
Wtot	Total work
