# Supplementary material for: Differential and Dose-Dependent Redistribution of Vitamin D Metabolites After Acute High-Intensity Exercise in Mixed Martial Arts Athletes and Untrained Men: Pilot Study
Source: Nutrients. 2026 Mar 26;18(7):1061. doi: 10.3390/nu18071061 (PMC13075115; doi:10.3390/nu18071061)
Supplement: Supplementary file 1 [file nutrients-18-01061-s001.zip › nutrients-4191803-supplementary.pdf]

## Supplementary data

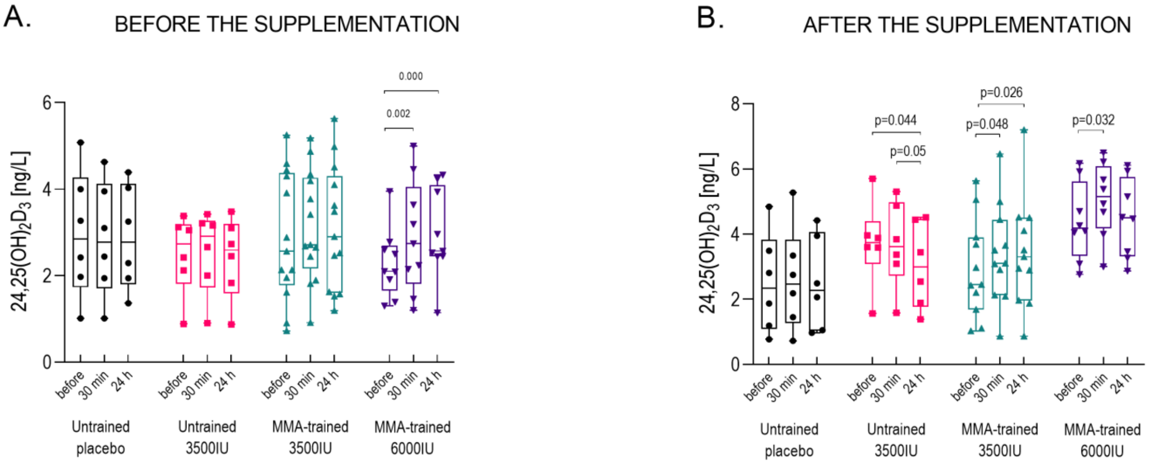


**Figure S1.** Acute exercise-induced changes in 24,25(OH)$_2$D$_3$. Serum 24,25(OH)$_2$D$_3$ concentrations measured before (**A**) and after supplementation (**B**) at baseline, 30 minutes, and 24 hours post-exercise in all experimental groups. Data are presented as box-and-whisker plots showing median and interquartile range (box: 25th–75th percentile), with whiskers indicating minimum and maximum values. Individual data points are displayed for each participant. Statistical analysis was performed using a two-way repeated-measures ANOVA (group × time), followed by LSD post hoc multiple-comparison testing.

**Table S1.** Mean power [W·kg$^{-1}$] obtained during Supramaximal Sprints

| Variable | Bout 1 | | | Bout 2 | | | Bout 3 | | |
|---|---|---|---|---|---|---|---|---|---|
| | BS Mean ± SD | AS Mean ± SD | p-value | BS Mean ± SD | AS Mean ± SD | p-value | BS Mean ± SD | AS Mean ± SD | p-value |
| Untrained-placebo | 7.96 ± 0.54 | 8.03 ± 0.36 | 0.647 | 5.74 ± 0.43 | 5.92 ± 0.55 | 0.403 | 4.60 ± 0.52 | 5.01 ± 0.45 | 0.222 |
| Untrained-3500 IU | 8.05 ± 0.36 | 8.54 ± 0.28 | 0.011 | 6.53 ± 0.52 | 6.64 ± 0.20 | 0.613 | 5.32 ± 0.59 | 5.59 ± 0.58 | 0.220 |
| MMA-trained 3500 IU | 7.98 ± 0.65 | 7.95 ± 0.48 | 0.784 | 6.45 ± 0.40 | 6.68 ± 0.45 | 0.143 | 5.41 ± 0.42 | 5.78 ± 0.53 | 0.137 |
| MMA-trained 6000 IU | 8.39 ± 0.64 | 8.62 ± 0.59 | 0.076 | 7.00 ± 0.49 | 7.27 ± 0.49 | 0.178 | 5.82 ± 0.89 | 6.35 ± 0.45 | 0.061 |

Values are presented as the mean ± SD; BS – before supplementation, AS – after supplementation.

**Table S2.** Total work [J·kg$^{-1}$] obtained during Supramaximal Sprints

| Variable | Bout 1 | | | Bout 2 | | | Bout 3 | | |
|---|---|---|---|---|---|---|---|---|---|
| | BS Mean ± SD | AS Mean ± SD | p-value | BS Mean ± SD | AS Mean ± SD | p-value | BS Mean ± SD | AS Mean ± SD | p-value |
| Untrained-placebo | 238.78 ± 16.34 | 240.92 ± 10.87 | 0.641 | 172.27 ± 12.88 | 177.53 ± 16.41 | 0.404 | 138.10 ± 15.56 | 150.35 ± 13.53 | 0.223 |
| Untrained-3500 IU | 241.48 ± 10.66 | 256.22 ± 8.34 | 0.011 | 195.82 ± 15.74 | 199.15 ± 6.05 | 0.622 | 159.75 ± 17.81 | 167.57 ± 17.36 | 0.221 |
| MMA-trained 3500 IU | 239.34 ± 19.46 | 238.37 ± 14.43 | 0.775 | 193.59 ± 11.86 | 200.51 ± 13.62 | 0.144 | 162.30 ± 12.7 | 173.44 ± 16.03 | 0.136 |
| MMA-trained 6000 IU | 251.61 ± 19.08 | 258.74 ± 17.59 | 0.716 | 209.97 ± 12.49 | 218.20 ± 14.82 | 0.176 | 174.57 ± 26.66 | 190.49 ± 13.51 | 0.061 |

Values are presented as the mean ± SD; BS – before supplementation, AS – after supplementation.

**Table S3.** Maximal power [W·kg$^{-1}$] obtained during Supramaximal Sprints

| Variable | Bout 1 | | | Bout 2 | | | Bout 3 | | |
|---|---|---|---|---|---|---|---|---|---|
| | BS Mean ± SD | AS Mean ± SD | p-value | BS Mean ± SD | AS Mean ± SD | p-value | BS Mean ± SD | AS Mean ± SD | p-value |
| Untrained-placebo | 10.38 ± 0.77 | 10.93 ± 0.45 | 0.018 | 7.96 ± 0.46 | 8.13 ± 1.32 | 0.988 | 5.90 ± 0.98 | 6.55 ± 0.77 | 0.185 |
| Untrained-3500 IU | 10.6 ± 0.76 | 10.99 ± 0.46 | 0.121 | 8.40 ± 0.71 | 8.69 ± 0.52 | 0.666 | 6.76 ± 0.92 | 7.47 ± 0.81 | 0.101 |
| MMA-trained 3500 IU | 10.47 ± 0.95 | 10.39 ± 1.22 | 0.631 | 8.65 ± 0.58 | 8.89 ± 0.92 | 0.389 | 7.38 ± 0.65 | 7.59 ± 1.06 | 0.552 |
| MMA-trained 6000 IU | 10.56 ± 1.08 | 10.85 ± 1.93 | 0.091 | 9.01 ± 0.84 | 9.66 ± 0.76 | 0.060 | 7.34 ± 1.38 | 8.42 ± 0.88 | 0.007 |

Values are presented as the mean ± SD; BS – before supplementation, AS – after supplementation.

**Table S4.** Time to maximal power [s] obtained during Supramaximal Sprints

| Variable | Bout 1 | | | Bout 2 | | | Bout 3 | | |
|---|---|---|---|---|---|---|---|---|---|
| | BS Mean ± SD | AS Mean ± SD | p-value | BS Mean ± SD | AS Mean ± SD | p-value | BS Mean ± SD | AS Mean ± SD | p-value |
| Untrained-placebo | 6.33 ± 1.84 | 4.97 ± 1.35 | 0.014 | 4.62 ± 1.05 | 5.28 ± 1.80 | 0.313 | 7.02 ± 2.45 | 7.07 ± 2.68 | 0.956 |
| Untrained-3500 IU | 5.61 ± 1.55 | 6.26 ± 2.82 | 0.120 | 5.37 ± 2.11 | 6.42 ± 2.06 | 0.104 | 8.47 ± 2.08 | 9.50 ± 2.10 | 0.090 |
| MMA-trained 3500 IU | 6.93 ± 2.02 | 6.38 ± 1.56 | 0.159 | 5.52 ± 1.27 | 5.56 ± 1.17 | 0.931 | 8.06 ± 1.97 | 7.50 ± 1.64 | 0.391 |
| MMA-trained 6000 IU | 5.63 ± 0.89 | 5.57 ± 1.06 | 0.909 | 5.32 ± 0.90 | 5.19 ± 0.88 | 0.802 | 7.34 ± 1.91 | 7.16 ± 1.66 | 0.676 |

Values are presented as the mean ± SD; BS – before supplementation, AS – after supplementation.
